# Older Adults as Covert Operatives in a First-Year Undergraduate Course on Aging

**DOI:** 10.1093/geroni/igaa057.1818

**Published:** 2020-12-16

**Authors:** Brian Carpenter, Meghan McDarby, Natalie Galucia, Nancy Morrow-Howell

**Affiliations:** 1 Washington University in St. Louis, St. Louis, Missouri, United States; 2 Washington University in St. Louis, Saint Louis, Missouri, United States

## Abstract

Age-friendly university programs are increasing in number, yet little research has evaluated how older adults shape classroom experiences. This pilot study tested one method for analyzing intergenerational classroom dynamics. Two small-group discussion sections for an introductory class on aging included older adults (n = 3 per section) and undergraduates (n = 15 per section). Class sessions on four topics (health, sexuality, housing, relationships) were video recorded. Overall, older adults spoke proportionally more during class discussions than would have been expected by chance alone. They participated most during the session about sexuality and least in the section that addressed relationships. Specific contributions from older adults included reflections on class activities, topic-specific personal anecdotes, and reactions to younger students. Research methods to investigate intergenerational learning are emerging, and this study provides one preliminary approach. We discuss additional ideas to bring empirical rigor to this emerging field of study.

